# Shifting paradigms: phosphatases from basic biology to druggable targets

**DOI:** 10.1242/bio.062400

**Published:** 2026-05-07

**Authors:** Catia L. Pierotti, Marian Brenner, Yamini Chand, Fabrice Krier, Melina Lappe, Franziska Schedel, Malgorzata Trebacz

**Affiliations:** ^1^ Signalling Research Centres BIOSS and CIBSS, University of Freiburg, Schänzlestraße 18, 79104 Freiburg im Breisgau, Germany; ^2^ Institute of Biology III, Faculty of Biology, University of Freiburg, Schänzlestraße 1, 79104 Freiburg im Breisgau, Germany; ^3^ Institute for Cell Biology, Department of Molecular Cell Biology, University of Bonn, Käthe-Kümmel-Straße 1, 53115 Bonn, Germany; ^4^ Institute of Pharmacology and Toxicology, University of Würzburg, Versbacher Straße 9, 97078 Würzburg, Germany; ^5^ Faculty of Chemistry and Pharmacy, Hermann-Staudinger Graduate School, University of Freiburg, Hebelstraße 27, 79087 Freiburg im Breisgau, Germany; ^6^ Spemann Graduate School of Biology and Medicine, University of Freiburg, Albertstraße 19A, 79104 Freiburg im Breisgau, Germany; ^7^ Anavo Therapeutics GmbH, Nikola-Tesla-Straße 1, 69124 Heidelberg, Germany

**Keywords:** Phosphatase, Workshop, Basic research, Translation, Phosphatase targeting, Cell signalling

## Abstract

The EMBO Workshop ‘Phosphatases: from basic research to translation’ (27 to 31 July 2025, Würzburg, Germany) brought together researchers from academia and industry for a deep dive into the rapidly evolving world of phosphatase biology. Over five days, participants explored the interplay of phosphatase structure, function, regulation, and signalling, while also uncovering their expanding influence in cancer, immunology, neurological disorders, and cardiometabolic health. The central focus of the meeting was the accelerating progress in phosphatase drug discovery, an enzyme class historically considered undruggable. This Meeting Review summarises the key findings and novel insights presented at the meeting and reflects the progress being made in the field.

## Introduction

Protein phosphorylation is the most abundant post-translational modification and plays a critical role in many, if not most, cellular processes including protein function and cell signalling. This reversible modification depends on the coordinated actions of two enzyme families: protein kinases, which add phosphate groups to proteins, and protein phosphatases, which hydrolyse phosphate groups from proteins. Although both kinases and phosphatases play essential roles in cellular functions, scientific research has generally focused more on kinases than phosphatases. However, phosphatases are increasingly being recognised by the scientific community as diverse and important enzymes, scaffolds, and signalling molecules involved in a plethora of cellular processes and human diseases, such as cancer, neurological, cardiometabolic and immune disorders. Despite being currently less well-characterised than their kinase counterparts, there is growing interest in phosphatases, from both a basic biology and a therapeutic targeting perspective, as highlighted by the emergence of phosphatase inhibitors in clinical trials. While phosphatases are now acknowledged as promising drug targets in a variety of human pathologies, significant gaps remain in our understanding of their molecular functions, structure, and regulation. This makes the phosphatase field an exciting and rapidly advancing area of research.

The 2025 EMBO Workshop on ‘Phosphatases: from basic research to translation’ was held from 27 to 31 July in Würzburg, Germany. It also featured a satellite meeting on 26 July, focused on phosphatase drug development from an academia and industry perspective, which was targeted at early-career researchers (ECRs). As one of the goals of the conference was to promote and support ECRs, this satellite meeting was specifically targeted at this group to promote their engagement, support their development, and create networking opportunities. The workshop and satellite meeting were organised by Antje Gohla (University of Würzburg, Germany) and co-organised by Maja Köhn (University of Bonn, Germany), Joe Lewis and Jacqueline Dreyer-Lamm (Anavo Therapeutics, Heidelberg, Germany and Leiden, Netherlands), Adrian Saurin (University of Dundee, UK), Mirela Delibegovic (University of Aberdeen, UK), and Jakob Nilsson (Danish Cancer Institute, Copenhagen, Denmark). This biennial workshop is an important international meeting in the field, and the only regular phosphatase conference in Europe. It provided a forum where scientists working on phosphatases meet to present and discuss recent developments in phosphatase research and identify the most important challenges that lie ahead, including substrate specificity, enzymatic regulation, availability of chemical tools, and translation of fundamental insights into therapeutic applications. The 2025 edition highlighted exciting, recent progress in the field, covering both fundamental questions about how phosphatases work at a molecular or cellular level, and translational questions about how best to target aberrant phosphatases driving diseases.

The conference showcased a stellar line-up of invited speakers and selected short talks from academia and industry, plus interactive poster sessions, meet-the-speakers and meet-the-editors sessions, and numerous networking opportunities at various social events. The programme provided a platform for scientific exchange, stimulating collaboration and building the phosphatase research community. Topics covered included phosphatases in cardio-metabolism, neurological disorders, cancer and the immune system, as well as phosphatase structure and biochemistry, targeting strategies, methods to study phosphatases and kinase/phosphatase modules in cellular signalling. These are key areas of interest not only in phosphatase research, but across biomedical research more broadly, which helps to position phosphatases as increasingly important players and therapeutically relevant contributors to these fields. The key outcomes from the conference emphasised how rapidly the field is developing and how these advances are redefining our understanding of the balance between kinases and phosphatases in signalling pathways, opening new opportunities for phosphatase research and clinical translation.

**Figure:**
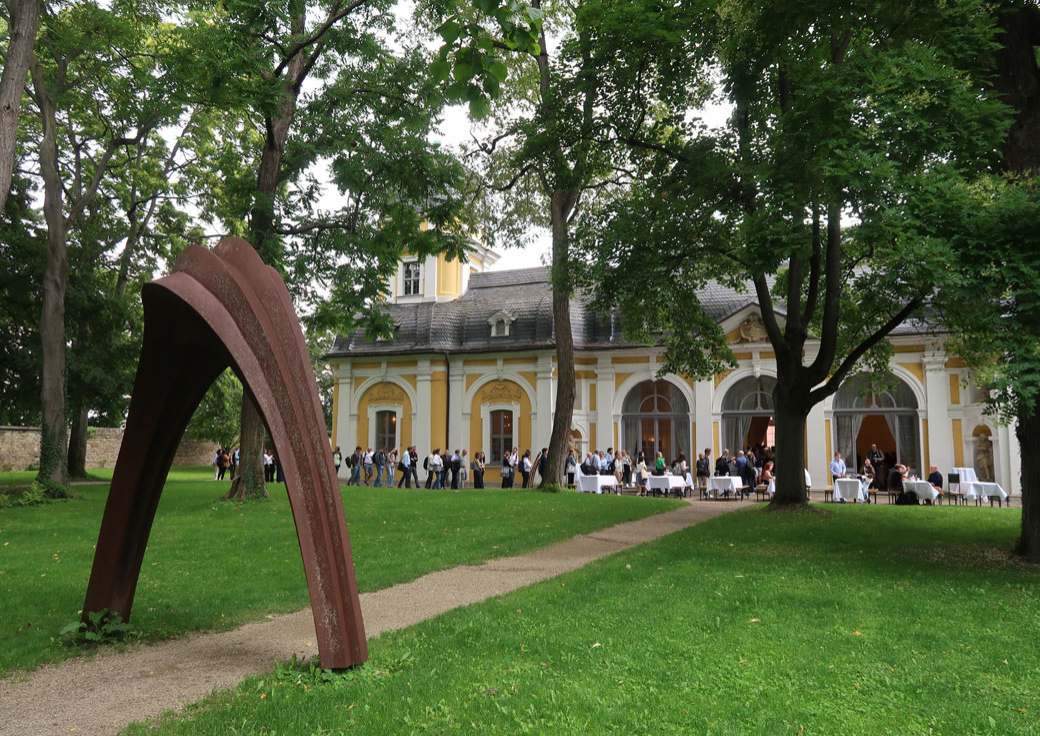
Participants at the EMBO Phosphatase Workshop during lunch at the Juliusspital Conference Centre in Würzburg, Germany.

**Figure:**
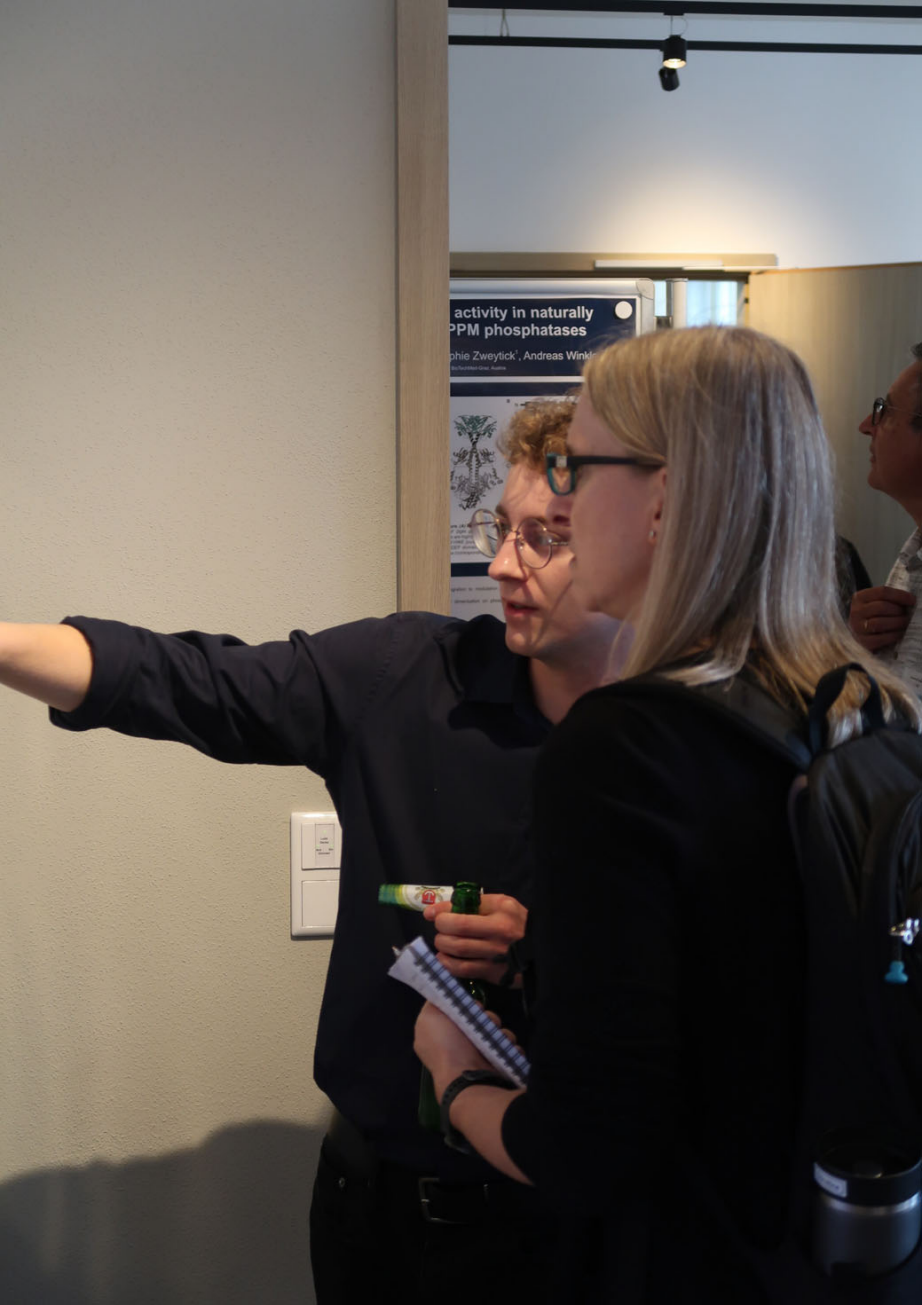
Keynote speaker Christina Baumgartner (AbbVie, Chicago, USA) and Marian Brenner (University of Würzburg, Germany) engaged in discussion during the poster session.

## Keynotes and new young investigators

The keynote lectures, opening and closing the meeting, highlighted two bodies of work that have made important contributions to the phosphatase field. Unlike kinases, for which numerous chemical inhibitors are used as therapeutics in the clinic, a major challenge in the phosphatase field has been the development of chemical inhibitors for therapeutic applications. In the opening keynote lecture, Christina Baumgartner (AbbVie, Chicago, USA) presented the small molecule ABBV-CLS-484, the first dual protein tyrosine phosphatase (PTP) inhibitor targeting PTPN2 (TC-PTP) and PTPN1 (PTP1B) to enter clinical trials, as a promising new approach for cancer immunotherapy (Baumgartner et al., 2023). This represents an important addition to the repertoire of phosphatase inhibitors entering clinical trials and demonstrates that, despite the challenges, developing drug candidates targeting phosphatases is indeed possible. In the closing keynote lecture, Martha Cyert (Stanford University, USA) presented a summary of her life's work studying calcineurin and discussed recent data on calcineurin signalling, introducing novel short linear motifs (SLiMs) and the discovery of calcimembrin, a membrane-targeting regulator of calcineurin (Ulengin-Talkish and Cyert, 2023; Bradburn et al., 2025). Calcineurin is a ubiquitous calcium-sensing phosphatase with important roles in calcium-dependent signal transduction and the target of immunosuppressant drugs, Cyclosporin A and FK506. These new insights into calcineurin regulation and signalling have important implications for the role of this phosphatase in human physiology and pathology.

As one of the goals of the meeting was to highlight the work of up-and-coming scientists in the phosphatase field, the meeting featured a young investigator session spotlighting the research of two recent principal investigators, who demonstrated the importance of phosphatase interacting partners for phosphatase function and therapeutic targeting. Stephanie Stanford (University of California, San Diego, USA) showed, in the context of targeting synovial fibroblasts in rheumatoid arthritis, the development of antibody-based biologics that block the binding site of glycosaminoglycan ligands and activate PTPRS to attenuate the disease phenotype (Svensson et al., 2020; Hein Da Rosa et al., 2025). Jasmeen Oberoi (University of Sussex, Brighton, UK) discussed the interaction between protein phosphatase 5 (PP5), the molecular chaperone heat shock protein 90 (HSP90), its co-chaperone CDC37 and its oncogenic kinase client BRAF^V600E^ by detailing the cryo-EM structures of HSP90:CDC37:BRAF^V600E^ complexes with either autoinhibited or activated PP5 (Oberoi et al., 2016, 2022). This session demonstrated the important discoveries that junior principal investigators are contributing to the phosphatase field.

## Cardio-metabolism

Protein phosphatases are key regulators of cardiovascular and metabolic signalling, and their dysregulation has been implicated in cardiovascular disease, obesity, insulin resistance and type 2 diabetes, among others (Bennett and Tiganis, 2025; Sun et al., 2024; Delibegovic et al., 2024). While the involvement of many phosphatases in these pathways and diseases is known, the precise mechanisms by which they act are often still unknown, and there is the additional challenge of targeting them without off-target effects. Speakers in this session contributed to furthering our understanding of the specific role that phosphatases play in cardio-metabolism, tackling major challenges in the field by giving insights into the mechanisms behind specific diseases or showing how recently developed inhibitors can specifically target single phosphatases in these diseases. Maria Kontaridis (Masonic Medical Research Institute, Utica, USA) opened the session by outlining a pathogenic role for Src homology 2 (SH2) domain-containing phosphatase 2 (SHP2) in systemic lupus erythematosus and showing that inhibition of this phosphatase can improve key features of the disease (Wang et al., 2016). Mirela Delibegovic (University of Aberdeen, UK) presented data on the pharmacological targeting of PTP1B in diabetic retinopathy, highlighting how modulation of this negative regulator of insulin and leptin signalling could influence retinal pathology (Forrester et al., 2020). Anton Bennett (Yale University School of Medicine, New Haven, USA), discussed the regulation of mitogen-activated protein kinase (MAPK) phosphatases (MKPs) and their links to disease, emphasising recent findings implicating MKP1 in non-alcoholic steatohepatitis (NASH) (Qiu et al., 2023). Gabriel Araujo (University Hospital Würzburg, Germany) reported that conditional loss of PP1β disrupts megakaryocyte maturation and platelet production through altered signalling pathways. To conclude the session, Stephanie Talamantes (Université Libre de Bruxelles, Brussels, Belgium) presented work on PTPN12, showing that changes in its abundance affect glucose metabolism and liver disease progression in steatohepatitis and diet-induced models of metabolic dysfunction (Talamantes et al., 2023). Overall, the session highlighted the progress in unveiling the specific involvement of phosphatases in cardio-metabolism, and the effect of their modulation, demonstrating their growing potential as targets for novel therapies.

## Neurological disorders

Protein phosphatases are increasingly recognised as critical but understudied regulators of brain function, yet major questions remain about how their dysregulation drives specific neurological disorders and whether they act independently of kinase imbalances. Once viewed mainly as passive counterparts to kinases, they are now emerging as genetically and mechanistically defined disease drivers (Hassan et al., 2024; Sandal et al., 2021). The speakers in this session addressed long-standing gaps in our understanding of how phosphatase mutations alter signalling networks and cellular organisation in the brain, providing new molecular insights that connect phosphatase dysfunction to both neurodevelopmental and neurodegenerative pathologies. Veerle Janssens (KU Leuven, Belgium) discussed protein phosphatase 2A (PP2A) mutations underlying Houge-Janssens syndrome (HJS). The mutations in the subtypes HJS1 and HJS4 prevent the PP2A:B56δ holoenzyme from binding Liprin α1, causing its hyperphosphorylation and aberrant liquid-liquid phase separation (LLPS) (Mayer et al., 2025). Matthew Gentry (University of Florida, Gainesville, USA) presented work on the phosphatase Laforin in Lafora disease, using spatial MALDI-IMS triple-omics to show how brain glycogen and N-glycan metabolism interconnect, with glycans shifting localisation in diseased versus wild-type tissue (Markussen et al., 2024). Extending these insights, Xin Zhang (Columbia University, New York, USA) reported that the catalytically inactive SHP2^C459S^ mutation – but not the activating SHP2^E76K^ – induces dysregulated GRB2-associated binding protein 1 (GAB1) phosphorylation in the presence of wild-type SHP2. His team rescued hydrocephalus in SHP2^C459S^ mice via allosteric SHP2 inhibition, linking the phenotype to SHP2 conformational changes (Makrides et al., 2025 preprint). Collectively, these presentations advanced the field by resolving key uncertainties about how individual phosphatase mutations translate into distinct neuronal phenotypes, offering functional evidence that reframes phosphatases as active contributors to disease mechanisms. By linking molecular changes to clinical outcomes and demonstrating therapeutic reversibility in model systems of neurological disorders, the session directly supported one of the meeting's central goals of integrating molecular discovery with translational strategies.

## Targeting strategies

The therapeutic targeting of phosphatases was a central theme of the workshop, representing one of the major challenges, but also one of the greatest opportunities, in the phosphatase field, and underscoring the importance of conferences as platforms for the exchange of ideas, new methodologies, and collaborative opportunities to progress in this area. Historically, phosphatases were considered undruggable for two main reasons. The first is their highly conserved, polar and positively charged catalytic sites, which often lack well-defined druggable pockets for small molecules (Ghattas et al., 2016). As a result, phosphatase inhibitors often require negatively charged functional groups to mimic phosphate substrates, leading to poor bioavailability. The second is that many serine/threonine phosphatases form complex holoenzyme assemblies with intricate and often constitutive regulatory mechanisms, further complicating efforts to selectively modulate their activity. This session provided insights into recent advances in this area, showing how these challenges can be overcome and how modulation of these enzymes may provide innovative strategies for treating a broad spectrum of diseases. Nicholas Tonks (Cold Spring Harbor Laboratory, USA) demonstrated that the PTP1B inhibitor DPM-1003 not only alleviated Rett syndrome symptoms in female methyl CpG-binding protein 2 (Mecp2)^+/−^ mice but also enhanced microglial metabolism, reduced amyloid-β levels, and ameliorated memory deficits in the amyloid precursor protein/presenilin 1 (APP/PS1) Alzheimer's disease mouse model (Krishnan et al., 2015; Cen et al., 2026 preprint). Building on this theme of precision targeting, Malgorzata Trebacz (Anavo Therapeutics, Heidelberg, Germany) presented the development of PTPN22 modulators, describing a dual-screening strategy that combined small molecule and fragment libraries with biochemical and structural data to generate potent ligands against this key autoimmune risk factor. Expanding the scope of therapeutic targeting of phosphatases, Zhong-Yin Zhang (Purdue University, West Lafayette, USA) discussed the mechanisms of oncogenicity of phosphatase of regenerating liver (PRL) family members, particularly PRL2, in tumourigenesis and outlined efforts to design covalent allosteric inhibitors of SHP1 for novel immunotherapy (Carlock et al., 2023). Concluding the session, Saurav Roy Choudhury (University of Toronto, Canada) identified a new skeletal muscle phosphatase as a novel link between inflammatory bowel disease and metabolic dysfunction, supported by findings from models of colitis, colitis-associated colon cancer, and obesity-related disease. Collectively, these studies underscore the growing progress in selectively targeting and modulating phosphatases across diverse pathological contexts, overcoming past difficulties by finding creative new approaches outside of targeting their active sites. The speakers provided insights into new strategies on finding selective modulators of phosphatase activity that could eventually lead to new compounds with improved pharmacological properties.

## Cancer and the immune system

While kinases often take the spotlight in cancer biology, this session highlighted how phosphatases are also important in maintaining homeostasis and driving disease when dysregulated. A phosphatase that plays an important role not only in cancer, but also in immune cells, is the tyrosine phosphatase SHP2. SHP2 mediates signalling downstream of cell surface receptors (Neel et al., 2003) and inhibitory immune cell receptors (Marasco et al., 2020), and can cause developmental disorders (Tartaglia et al., 2001) or promote cancer (Mohi and Neel, 2007) when mutated, depending on alterations in conformation and activity. However, its enzymatic versus non-enzymatic (i.e. scaffolding or adaptor) functions, regulation and the influence of specific phosphorylation sites remain incompletely understood. Björn Lillemeier (University of Freiburg, Germany) presented a combination of *in vitro* assays and microscopy approaches to demonstrate how phosphorylation of different tyrosine sites on SHP2 can modulate its catalytic activity. Benjamin Neel (NYU Langone Health, New York, USA) revealed how the combination of catalytic activity and structural regulation controls SHP2 function in different cellular contexts. These two speakers provided novel insights into SHP2 function, regulation and phosphorylation that address knowledge gaps in the SHP2 field.

Another phosphatase with an important role in cancer is the serine/threonine phosphatase PP2A, which acts as a tumour suppressor. Rosalie Sears (Oregon Health and Science University, Portland, USA) focused on PP2A and its relationship to MYC in pancreatic disease, describing how shifts in PP2A subunit composition during inflammation and tumour progression can alter MYC stability and how understanding these switches could open new therapeutic possibilities (Allen-Petersen and Sears, 2019; Tinsley et al., 2024). Furthermore, a re-evaluation of the anti-cancer compound DT-061 by Ingrid Frohner (Medical University of Vienna, Austria), extended its effects beyond PP2A activation to broader pathways, reshaping how such agents might induce cancer cell death. Her talk was a useful reminder that careful biochemical validation for the interpretation of drug action requires orthogonal assays and attention to cell state. Also focusing on phosphatases in cancer research, Ahmed Elbatsh (Novartis Institute for Biomedical Research, Basel, Switzerland) showed that a CRISPR-Cas9 screen identified inositol polyphosphate 5-phosphatase A (INPP5A) as a key dependency in uveal melanoma cells carrying GNAQ or GNA11 mutations. He demonstrated that the link between INPP5A loss and GNAQ/11 mutations opens new therapeutic opportunities for this most lethal melanoma subtype (Elbatsh et al., 2024). Together, these talks contributed novel avenues for cancer therapeutics by harnessing phosphatase-driven mechanisms.

Phosphatases also have a multitude of functions in the immune system. The roles of the related tyrosine phosphatases PTP1B and TC-PTP, emphasising their similarities and differences, were introduced by Michel Tremblay (McGill University, Montreal, Canada) who presented applications of their genetic modulation and small-molecule inhibition in cell therapeutics and systemic immunotherapy (Perez-Quintero et al., 2024). This topic was continued by Tony Tiganis (Monash University, Clayton, Australia) who described how intracellular phosphatases should be considered immune checkpoints similar to extracellular targets such as programmed cell death protein 1 (PD-1)/programmed cell death ligand 1 (PD-L1). He demonstrated that deletion of TC-PTP in T cells markedly enhances anti-tumour immunity (Wiede et al., 2020; Goh et al., 2022), and similarly, that PTP1B deletion or inhibition enhances T cell expansion and cytotoxicity, improving the efficacy of CAR T cell therapy (Wiede et al., 2022). Together, these two speakers established, using genetic validation and chemical inhibition, that TC-PTP and PTP1B are promising targets in immunotherapy. In addition, Anna Lippert (University of Würzburg, Germany) offered a biophysical perspective on how the localisation of phosphatases and steric effects influence receptor activation, shedding new light on how membrane organisation establishes signalling thresholds in immunity and cancer, as well as providing new strategies for antibody design (Lippert et al., 2024). Altogether, this session highlighted the important roles of phosphatases in oncology and immunology, and demonstrated how the conference served as a platform for the exchange of ideas and discussions to advance in these research areas.

## Structure and biochemistry

Some of the key unanswered questions in the phosphatase field concern substrate recognition and specificity, structural regulatory mechanisms, and evolution and diversification. This session provided the phosphatase community with the opportunity to learn about and discuss the latest contributions towards better understanding the structural and biochemical principles that govern phosphatase substrate recognition, activity regulation, and functional evolution. Wolfgang Peti (University of Connecticut Health Center, Farmington, USA) opened with substrate specificity in serine/threonine phosphatases, using calcineurin as a model. He emphasised that activity depends not only on the conserved catalytic core but also on dynamic charge-charge contacts in intrinsically disordered regions, known as ‘fuzzy’ interactions. Both ordered and disordered SLiMs were shown to facilitate enzyme assembly and substrate recruitment (Katti et al., 2025). Expanding on functional diversification, Thomas Leonard (Max Perutz Labs, Vienna, Austria) presented work on Pleckstrin homology (PH) domain leucine-rich repeat-containing protein phosphatase 2 (PHLPP2), demonstrating that it lacks catalytic activity *in vitro* although it may have retained ancestral substrate binding as indicated by surface conservation analysis. Phylogenetic analysis determined that PHLPP2 lost catalytic function at the base of the metazoan lineage and suggested a scaffolding role at membranes rather than direct enzymatic function (Husremović et al., 2025).

The serine/threonine phosphatase PP2A has important functions in cell cycle regulation. Arminja Kettenbach (Geisel School of Medicine at Dartmouth, Hanover, USA) further illustrated regulatory adaptation by examining the PP2A-B55 subunit in the cell cycle. Using quantitative (phospho-) proteomics, her team identified unique PP2A-B55 and PP1 dephosphorylation sites and showed that PP2A-B55 reactivation at mitotic exit depends on protein degradation (Wasserman et al., 2024). Paula Sotelo-Parrilla (Ludwig Maximilian University of Munich, Germany) detailed structural studies of PP2A-B56 interactions with Shugoshin 1 and 2, which are essential for PP2A-B56 recruitment to centromeres and kinetochores during mitosis. By integrating structural biology, biophysics, and cell biology, she defined molecular interactions critical for chromosome segregation (Ueki et al., 2021). Collectively, these talks addressed knowledge gaps in our understanding of how phosphatases function at a structural and molecular level. Importantly, they illustrated how conserved catalytic frameworks are diversified through disordered motifs, regulatory subunits, and evolutionary adaptation, revealing the structural mechanisms that fine-tune phosphatase function in cellular processes.

## Methods to study phosphatases

One persistent challenge in phosphatase research has been the relative lack of tools compared to those available for kinases. This session highlighted how researchers are addressing this gap through innovative experimental, computational, and chemical strategies. Key questions addressed in this session included how to study phosphatases in their cellular context, how to interpret the complex datasets that such approaches generate, and how to access areas of phosphate metabolism that remain incompletely characterised due to limited detection methods. By combining global phosphoproteomics with functional studies, Bettina Warscheid (University of Würzburg, Germany) showed that the actin-binding protein filamin C (FLNc), acts as a central hub in skeletal muscle stress responses, and how a finely tuned kinase-phosphatase balance maintains FLNc stability and supports myofibril repair (Kokot et al., 2024). This kind of systems-level view is increasingly important, but it generates enormous, complex datasets. Evangelia Petsalaki (EMBL-EBI, Hinxton, UK) provided a computational perspective, introducing three complementary tools for making better use of (phospho)proteomics datasets to study cell signalling: phuEGO (Giudice et al., 2024) for cleaning noisy data, SELPHI2 (Maier et al., 2025) for predicting regulatory connections, and PhosX (Lussana et al., 2024) for measuring upstream activity. These approaches illustrate how experimental and computational methods are advancing together to make sense of complex phosphorylation networks. The meeting also featured methods for studying phosphate-containing molecules. Firstly, from a chemical tools perspective, Henning Jessen (University of Freiburg, Germany) presented FRET-polyP8, a tool to study enzymes that break down polyphosphates (polyP), which led to the discovery of new manganese-dependent endopolyphosphatase activity in yeast (Moser et al., 2025). Furthermore, Dorothea Fiedler (Leibniz-FMP, Berlin, Germany) introduced a ^13^C isotopic labelling and NMR approach for studying inositol phosphates and pyrophosphates (InsPs/PP-InsPs), which revealed a previously unrecognised multiple inositol polyphosphate phosphatase 1 (MINPP1)-dependent pathway in human cells and identified new InsP kinases and phosphatases (Patharkar et al., 2025). Both talks underscored how developing better tools and detection methods opens up areas of phosphate metabolism that have remained incompletely characterised. Looking toward applications, Po-Han Chen (National Cheng Kung University, Tainan, Taiwan) described phosphorylation-targeting chimeras (PhosTACs), which recruit phosphatases to specific substrates, demonstrating targeted dephosphorylation of tau in Alzheimer's disease (Hu et al., 2023), α-synuclein in Parkinson's disease (Chen et al., 2025), and epidermal growth factor receptor (EGFR) in cancer (Hu et al., 2024). Together, these talks highlighted the importance of robust chemical tools, computational methods and experimental approaches to advance phosphatase research and therapeutic opportunities, and collectively demonstrated how the field is moving from data generation toward mechanistic understanding and, ultimately, therapeutic application.

## Cellular signalling

While phosphatases are now widely recognised as key regulators of signalling pathways in many cellular functions, many outstanding questions remain regarding precisely how certain phosphatases modulate specific signalling events. These include their specific substrates, regulators and interactors within a given signalling pathway, as well as the effects of spatial organisation, pathway crosstalk and signalling dynamics. This session spotlighted recent efforts to tackle some of these outstanding questions, highlighting how phosphorylation events fine-tune dynamic signalling networks and showcasing emerging technologies that enable detailed analyses of these processes. The EMBO Young Investigator Programme (YIP) lecture by Hayley Sharpe (Babraham Institute, Cambridge, UK) revisited the widely used ‘substrate trapping’ methods for protein tyrosine phosphatases. Her findings showed that pervanadate acts far beyond blocking PTP catalytic activity, promoting widespread cysteine oxidation across the proteome. This has broad implications for tyrosine phosphorylation signalling (Mulholland et al., 2025). New insights on the myosin phosphatase/protein arginine methyltransferase 5/histone pathway were presented by Beata Lontay (University of Debrecen, Hungary) who provided evidence that protein phosphatase 1B (PPM1B) modulates this oncogenic pathway. Her findings show that PPM1B functions as a tumour suppressor and may serve as a potential biomarker for cervical carcinoma (Keller et al., 2025). While signalling proteins are usually limited by random diffusion when recruited to activated receptor tyrosine kinases, LLPS can assist by concentrating proteins. John Ladbury (University of Leeds, UK) showed that SHP2 dynamically modulates receptor activity by acting as a scaffold to organise protein assemblies and mediate downstream signalling. This study exemplifies how a single multifunctional phosphatase can fine-tune cellular responses and exert significant influence on disease-related pathways (Ladbury et al., 2023; Lin et al., 2022).

Phosphatases also regulate signalling pathways and biological processes in the nucleus of cells. Giacomo Cossa (University of Würzburg, Germany) highlighted the PP1-PNUTS complex as a key modulator of RNA polymerase II, revealing a previously unrecognised role for PNUTS, a regulatory interactor of protein phosphatase 1 (RIPPO) (Cossa et al., 2020). Anne Donaldson (University of Aberdeen, UK) provided insights into DNA replication control, elucidating the mechanism by which Rif1 functions as a RIPPO (Garzón et al., 2019; Isobe et al., 2021). Anna Castro (Université de Montpellier, France) examined PP2A-B55 phosphatase regulation during meiosis and highlights Greatwall (Gwl) as a pivotal regulator that safeguards meiotic fidelity (Lacroix et al., 2024; Roumbo et al., 2025). These findings demonstrated the broad scope of phosphatase mediated signalling and showed how the conference provided a platform to discuss the wide variety of cellular processes regulated by phosphatases.

A mechanistic understanding of cellular signalling requires not only information on the phosphorylation status of individual proteins, but also precise data on the specific residues modified and their temporal dynamics. To address this requirement, new tools for investigating phosphorylation processes were presented by Jakob Nilsson (Danish Cancer Institute, Copenhagen, Denmark) and Adrian Saurin (University of Dundee, UK). Nilsson introduced a pooled base editor sensor screening method that enables systematic mutation of phosphorylation sites. This powerful approach enables the identification of previously overlooked but functionally critical phosphorylation sites within major signalling pathways (Ambjoern et al., 2026; Meeusen et al., 2025 preprint). Saurin, in turn, developed a powerful mass spectrometry-based approach to measure the dynamics of protein phosphorylation (Allan et al., 2025; Gelens and Saurin, 2018). Overall, this session emphasised the dynamic and multifaceted roles that phosphatases play in cellular regulation, extending far beyond simple dephosphorylation. Their activities are tightly orchestrated within signalling networks through complex regulatory mechanisms, allowing fine-tuned control of cellular responses and highlighting their importance in both physiological processes and disease contexts.

## Poster sessions and prize winners

The workshop featured two poster sessions, with 61 posters presented. All posters were presented in flash talks preceding the poster sessions. Participants came from around the world, ranging from PhD students and postdoctoral researchers to principal investigators and scientists working in industry. This highlights, not only the diversity of phosphatase research at the meeting, but also the diversity of attendees in terms of their career stage, place of work and geographical location.

### Supporting ECRs

A core objective of the workshop was to support and promote ECRs, recognising their essential role in advancing phosphatase research. To achieve this goal, extensive support was provided through EMBO travel grants, registration fee waivers, and childcare grants that enabled many ECRs to participate. The programme included dedicated networking opportunities through academia-industry pub sessions specifically designed for ECRs, as well as ‘Meet the Speaker’ and ‘Meet the Editors’ sessions that provided valuable mentoring opportunities.

### Prize winners

Eight prizes were awarded to recognise outstanding posters and scientific excellence, with awards helping to support ECR travel costs. The winning posters reflected the breadth of the phosphatase field, spanning immune cell signalling, cancer biology, structural mechanisms of phosphatase regulation, and muscle physiology:

- Katarzyna Wojdyla (Babraham Institute, Cambridge, UK) – “Understanding the CD45 signalling network in immune cells”
- Ádám Ungvári (University of Debrecen, Hungary) – “Mg²^+^/Mn²^+^-dependent protein phosphatase 1B plays a crucial role in cervical cancer through the regulation of myosin phosphatase/protein arginine methyltransferase 5/histone 4 oncogenic axis”
- Shruti Bhardiya (University of Turku, Finland) – “Targeting DUSP6 to combat therapy resistance and brain metastasis in HER2-positive breast cancer”
- Drshika Mehtani (Babraham Institute, Cambridge, UK) – “Redox mediated substrate interactions with PTPRK”
- Bob Meeusen (University of Copenhagen, Denmark) – “A functional map of phosphoprotein phosphatase regulation identifies an evolutionary conserved reductase for the catalytic metal ions”
- Ingrid Frohner (Max Perutz Labs, Vienna, Austria) – “Molecular mechanism of the anti-cancer drug DT-061”
- Björn Brinschwitz (University Medicine Greifswald, Germany) – “T-cell protein tyrosine phosphatase (TCPTP) and protein tyrosine phosphatase 1B (PTP1B) restrict the interferon γ and NLRP3 response in myocytes and protect from sepsis-induced muscle wasting”
- Andrea Corno (University of Dundee, UK) – “A chemical-genetic system to rapidly inhibit the PP2A-B56 phosphatase reveals a role at metaphase kinetochores”

## Conclusion

The EMBO Phosphatase Workshop provided valuable insights into the recent advances and challenges in the phosphatase field. It impactfully contributed to the progress of phosphatase research by helping to define emerging challenges, encouraging innovation in concepts and approaches to study phosphatases, and highlighting the expanding importance of phosphatases in cell signalling. The conference brought together the community to discuss the most current advances in the field across cell biology, structural insights, regulatory mechanisms, and emerging strategies for drug targeting. By connecting researchers from diverse disciplines and career stages, the conference promoted scientific dialogue, supported early-career investigators, and strengthened relationships between academic and industrial partners. Importantly, the workshop showcased a paradigm shift – from viewing phosphatases as purely academic interests to recognising them as viable and promising drug targets. Despite recent advances, significant challenges persist, particularly in delineating substrate specificity and elucidating the context-dependent regulation of phosphatase activity across diverse cellular environments. The workshop emphasised the importance of collaboration between academia and industry to accelerate the translation of basic discoveries into clinical applications. This shift in perspective is setting the stage for a productive exchange of ideas and a clear direction for future research and translational efforts in the phosphatase field.
